# Supplementary material for: Glucose Favors Lipid Anabolic Metabolism in the Invasive Breast Cancer Cell Line MDA-MB-231
Source: Biology (Basel). 2020 Jan 10;9(1):16. doi: 10.3390/biology9010016 (PMC7168317; doi:10.3390/biology9010016)
Supplement: Supplementary file 1 [file biology-09-00016-s001.pdf]

Supplementary Figures

Glucose favors lipid anabolic metabolism in the invasive breast cancer cell line MDA-MB-231

Mª Carmen Ocaña [1,2], Beatriz Martínez-Poveda [1,2], Ana R. Quesada [1,2,3] and Miguel Ángel Medina [1,2,3,*]

[1] Universidad de Málaga, Andalucía Tech, Departamento de Biología Molecular y Bioquímica, Facultad de Ciencias, E-29071 Málaga, Spain
[2] IBIMA (Biomedical Research Institute of Málaga), E-29071 Málaga, Spain
[3] CIBER de Enfermedades Raras (CIBERER), E-29071 Málaga, Spain
* Correspondence: medina@uma.es; Tel.: +34 952137132

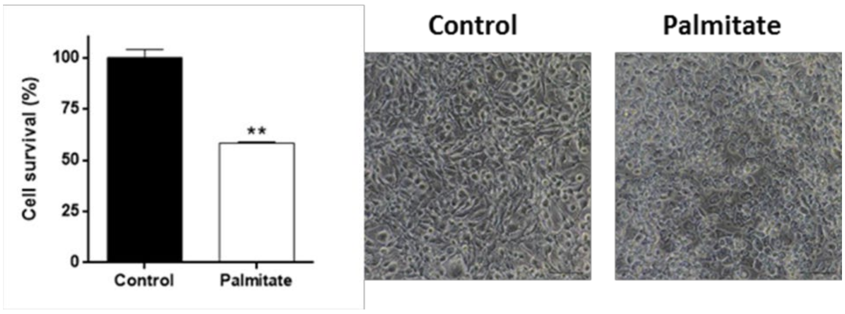

Figure S1. Effect of long time exposure with palmitate in MDA-MB-231 cells. Representative photographs and quantification of 0.5 mM palmitate effect on cell viability in media containing glucose and glutamine after an overnight incubation. Bar scale = 91.75 μm. Data are expressed as means ± SD of one experiment with duplicates. **$p < 0.01$.
